# Microlearning in Health Professions Education: Scoping Review

**DOI:** 10.2196/13997

**Published:** 2019-07-23

**Authors:** Jennie Chang De Gagne, Hyeyoung Kate Park, Katherine Hall, Amanda Woodward, Sandra Yamane, Sang Suk Kim

**Affiliations:** 1 School of Nursing Duke University Durham, NC United States; 2 School of Nursing West Virginia University Morgantown, WV United States; 3 Duke Medical Center Library Durham, NC United States; 4 Department of Nursing Catawba College Salisbury, NC United States; 5 Red Cross College of Nursing Chung-Ang University Seoul Republic of Korea

**Keywords:** online learning, health occupations students, mobile applications, active learning, distance learning, Web 2.0

## Abstract

**Background:**

Microlearning, the acquisition of knowledge or skills in the form of small units, is endorsed by health professions educators as a means of facilitating student learning, training, and continuing education, but it is difficult to define in terms of its features and outcomes.

**Objective:**

This review aimed to conduct a systematic search of the literature on microlearning in health professions education to identify key concepts, characterize microlearning as an educational strategy, and evaluate pedagogical outcomes experienced by health professions students.

**Methods:**

A scoping review was performed using the bibliographic databases PubMed (MEDLINE), CINAHL, Education Resources Information Center, EMBASE, PsycINFO, Education Full Text (HW Wilson), and ProQuest Dissertations and Theses Global. A combination of keywords and subject headings related to microlearning, electronic learning, or just-in-time learning combined with health professions education was used. No date limits were placed on the search, but inclusion was limited to materials published in English. Pedagogical outcomes were evaluated according to the 4-level Kirkpatrick model.

**Results:**

A total of 3096 references were retrieved, of which 17 articles were selected after applying the inclusion and exclusion criteria. Articles that met the criteria were published between 2011 and 2018, and their authors were from a range of countries, including the United States, China, India, Australia, Canada, Iran, Netherlands, Taiwan, and the United Kingdom. The 17 studies reviewed included various health-related disciplines, such as medicine, nursing, pharmacy, dentistry, and allied health. Although microlearning appeared in a variety of subject areas, different technologies, such as podcast, short messaging service, microblogging, and social networking service, were also used. On the basis of Buchem and Hamelmann’s 10 microlearning concepts, each study satisfied at least 40% of the characteristics, whereas all studies featured concepts of maximum time spent less than 15 min as well as content aggregation. According to our assessment of each article using the Kirkpatrick model, 94% (16/17) assessed student reactions to the microlearning (level 1), 82% (14/17) evaluated knowledge or skill acquisition (level 2), 29% (5/17) measured the effect of the microlearning on student behavior (level 3), and no studies were found at the highest level.

**Conclusions:**

Microlearning as an educational strategy has demonstrated a positive effect on the knowledge and confidence of health professions students in performing procedures, retaining knowledge, studying, and engaging in collaborative learning. However, downsides to microlearning include pedagogical discomfort, technology inequalities, and privacy concerns. Future research should look at higher-level outcomes, including benefits to patients or practice changes. The findings of this scoping review will inform education researchers, faculty, and academic administrators on the application of microlearning, pinpoint gaps in the literature, and help identify opportunities for instructional designers and subject matter experts to improve course content in didactic and clinical settings.

## Introduction

### Background

Technology is changing the way the world communicates—how we learn, remember, and transform information [1]. Many health disciplines, such as allied health, dentistry, medicine, nursing, and pharmacy, are embracing emerging technologies to leverage learning opportunities for their students [2]. One such innovative pedagogy is the practice of microlearning, which refers to small lesson modules and short-term activities intended to teach and reinforce course objectives [3]. One of the advantages of this pedagogy is the asynchronistic aspect, allowing the learner to control the place, method, and time of access to information [3]. Although microlearning is characterized in terms of the size of content, learning occurs remarkably quickly within minutes or seconds of time instead of hours, days, or months, a concept known as *just-in-time* learning [4,5]. Although microlearning is an emerging trend especially in continuing education [4,5], no standardized concepts or applications have been established in health professions education.

Referred to as *micro-* or *bite-sized content*, *microcourses*, or *just-enough information*, microlearning teaches a small learning unit in a step-by-step approach [3,5]. The emergence of user-generated content such as Web 2.0 has enabled participants to generate large amounts of information that can be circulated immediately worldwide [6]. Microlearning harnesses Web 2.0 technologies to engage students and to promote self-determined learning, also known as heutagogy [7,8]. This learning theory emphasizes the creativity, flexibility, and ability of learners [9]. It empowers students to be self-directed and self-determined in their own learning [7,10]. The ubiquitousness of Web 2.0 has contributed to the renewed attention to heutagogy [7], a learner-centric approach that enables students to access smaller, targeted, and manageable chunks of information available on the Web at their convenience [4,11]. In contrast to reading chapters in a textbook and memorizing content as in older education designs, microlearning is more favorable than macrolearning to students in that the former encourages students to attain information that is as up to date as possible in the moment they are ready or need to learn the material, whereas the latter is usually organized in a hierarchical and static manner [3,11].

As the amount of information that learners are faced with has increased, microlearning can help break down the material into smaller units that can be processed more easily [2,8,11]. Learning is then focused on making connections between and among the small units, which is a foundation of critical thinking and clinical reasoning [3,11]. This is particularly important in health professions education, which changes constantly with advancements in medicine and health care delivery systems [12]. The effectiveness of microlearning for health care professionals has been reported in clinical studies, such as a mobile app for recording learning experiences in nursing practice [13]; an interactive case-based teaching session in medical training programs [14]; a mobile gaming device that promotes nursing research knowledge, attitudes, and practice [15]; and a streaming video system with point-of-view camera transmission of surgeries to students’ smartphones and tablets [16]. As such, microlearning has been endorsed by many health professions educators, programs, and organizations as a means of facilitating student learning, training, and continuing education [17,18].

### Objectives

Despite its popularity and applicability to a wide range of health disciplines, microlearning is difficult to define in terms of its features and processes [5,19]. Moreover, a systematic review has not previously been used to analyze studies on health professions students’ microlearning and the outcomes associated with this pedagogy. Thus, the purpose of this review was to (1) conduct a systematic search of the literature on microlearning utilized for health professions students to identify key concepts and gaps in the research, (2) describe the nature of educational outcomes associated with microlearning experienced by health professions students, and (3) examine how microlearning was characterized as an educational strategy for health professions students.

## Methods

### Framework

This scoping review follows the Joanna Briggs Institute (JBI) methodology to map the key concepts of microlearning within health professions education. In contrast to systematic reviews that strive to answer a precise question, scoping reviews are designed to determine the extent and nature of the evidence available on a topic [20]. To facilitate this broader scope, the objectives of this review were developed using the Population-Concept-Context model, where the population is health professions students, the concept is microlearning, and the context is any learning environment where microlearning was introduced and evaluated. This review was conducted following the 5-step framework by Arksey and O’Malley: (1) identify the research questions; (2) identify the relevant studies; (3) select studies; (4) chart or map the data; and (5) collate, summarize, and report the data [21]. The Preferred Reporting Items for Systematic Reviews and Meta-Analyses extension for Scoping Reviews checklist guided the reporting of this review [22]. A protocol for the review was published in the *JBI Database of Systematic Reviews and Implementation Reports* [23].

### Search Strategy

The search strategy was developed to comprehensively identify published and unpublished literature following the 3-step approach developed by JBI [20]. First, a preliminary search was conducted in PubMed (MEDLINE) and CINAHL. The authors analyzed the titles, abstracts, and index terms used to describe the articles captured in the initial search. This informed the second phase of the search process where the strategy was finalized and then tailored to each information source. The search was conducted in PubMed (MEDLINE), CINAHL, Education Resources Information Center, EMBASE, PsycINFO, Education Full Text (HW Wilson), and ProQuest Dissertations and Theses Global using a combination of keywords and subject headings related to microlearning, electronic learning (e-learning), or just-in-time learning combined with health professions education. No date limits were placed on the search, but the results were limited to English-language materials. A full search strategy for each database is detailed in Multimedia Appendix 1. The search was conducted from January to May 2018. After full-text screening of the search results, the third phase of the search process involved reviewing reference lists for articles.

### Study Selection

All identified citations were managed using EndNote V8.2 (Clarivate Analytics), and duplicates were removed. The citations were imported into the Covidence systematic review software (Veritas Health Innovation) for title and abstract screening by 2 independent reviewers. Studies were included if the following criteria were met: (1) they reported on concepts of microlearning in the form of micro- or bite-sized content, microcourses, *just-in-time* learning, or just-enough information; (2) they involved health professions students, defined as undergraduate medical students, prelicensure medical students, undergraduate or graduate nursing students, dentistry students, pharmacy students, and allied health professions students; and (3) they took place in an academic setting, hospital training setting, community learning setting, clinical skills laboratory, virtual class, or any other setting where microlearning in health professions was introduced and evaluated. The full texts of selected studies were retrieved and assessed in detail against the inclusion criteria. Full-text studies that did not meet the inclusion criteria were excluded, and the reasons for exclusion were noted. Disagreements between the reviewers were resolved through discussion or with a third reviewer.

### Data Extraction and Synthesis

Data were extracted from eligible publications included in the review using the standardized data extraction tool in Covidence. NVivo 12 (QSR International Pty Ltd) was also used to assist in organizing, synthesizing, and identifying emerging themes from the literature review. In this method, each document stored in EndNote was imported into NVivo as a case, and the case was then assigned attributes (ie, study year, author(s), country, study purposes, study design, target audience, sample size, theoretical framework, definition of microlearning, course or instructional design, topic, technology platform, measurement tool, key findings, and learning outcomes). Pedagogical outcomes were assessed according to Kirkpatrick 4 levels of evaluation (reaction, learning, behavior, and results)—the most widely used program evaluation strategy in both traditional classrooms and mobile learning in health professions education [24,25]. A formal assessment of methodological quality was not performed as this scoping review aimed to provide an overview of the existing evidence regardless of quality [20].

## Results

### Overview

Our search yielded 3096 potentially relevant studies. Of the 246 articles that underwent full-text review, 229 (93.1%) were excluded for the following reasons: absence of the concept of microlearning (103/229, 45.0%), conference abstracts (65/229, 28.4%), nonempirical literature such as review or editorials (22/229, 10.0%), focused on the evaluation of technology rather than learning (19/229, 8.3%), only available in abstract form (7/229, 3.1%), did not target health professions education (6/229, 3.0%), duplicate article (4/229, 1.7%), or non-English literature (3/229, 1.3%). Ultimately, 17 articles met the inclusion criteria (Multimedia Appendix 2).

### Study Characteristics

The 17 studies reviewed included 2228 participants in various health-related disciplines such as medicine (n=8), nursing (n=3), pharmacy (n=2), dentistry (n=2), and allied health (n=2). The course topics that were taught via microlearning included violence response, graduate psychology, splinting techniques, pharmacology, public health, embryology, dentistry, physiomics, internal medicine clerkship, biochemistry, cellular biology, anatomy and physiology, urology, mental health, and pharmacotherapy. Articles that met the inclusion criteria were published in 2011 through the first half of 2018, which reflects the contemporary practice of microlearning and research interest in the subject.

The researchers came from a wide range of countries, including the United States (n=5), China (n=3), India (n=3), Australia (n=1), Canada (n=1), Iran (n=1), the Netherlands (n=1), Taiwan (n=1), and the United Kingdom (n=1). Research methods included quasi-experimental (n=6), mixed methods (n=5), descriptive (n=4), randomized control trial (n=1), and correlation (n=1). Many different technology platforms and apps were also utilized, including podcast (n=7), short messaging service (SMS; n=4), microblogging (n=3), social networking service (n=2), and internet-based apps (n=1). Interestingly, none of the 17 studies provided a definition of microlearning.

### Kirkpatrick Outcome Evaluation

Pedagogical outcomes were assessed according to Kirkpatrick 4 levels of evaluation [25]. Level 1 is *reaction,* where learners react to the learning event with a positive attitude such as satisfaction or engagement. Level 2 is *learning,* where learners obtain knowledge, skills, confidence, and commitment by engaging in the learning event. Level 3 is *behavior,* where learners apply their acquired knowledge, skills, confidence, and commitment to real tasks such as practical examinations or final course grades. Level 4 is *results* where learners provide benefit to the patients or practice, such as patient safety or quality of care, utilizing those acquired knowledge, skills, confidence, or commitment [25].

Reaction, one of the easiest outcomes to measure, can be determined by evaluating the user’s engagement, relevance, and satisfaction [25]. Overall, 16 out of 17 studies (94%) in our review measured student responses to the microlearning interventions with qualitative assessments. For example, Ball et al gathered student feedback on the helpfulness, relevance, and appropriateness of video podcasts [26], whereas Bledsoe et al gathered student opinions on access to information, communication, classroom engagement, and overall experience with the Twitter learning intervention [27]. Learning, level 2 in Kirkpatrick model can be evaluated based on the knowledge, skills, attitudes, confidence, and commitment that participants gain [25]. In total, 14 of the 17 studies (82%) evaluated student learning this way. For example, Evans evaluated student achievement in an end-of-module written examination that demonstrated high average levels of knowledge and understanding [28]. Similarly, Cheng et al measured students’ success of splint application against a 6-point skills checklist and then compared the completion times among the groups [29].

Behavior, level 3, is considered the most important outcome to assess because it is the degree to which learners will apply what they have learned when they are practicing on their own [25]. Overall, 5 of the 17 studies (29%) measured student behavior. Among them, Diug et al examined the long-term effects of Twitter learning interventions by comparing students’ end-of-semester grades [30]. Lameris et al evaluated the effect of their smartphone app on student behavior by comparing the number of hours students spent studying before and after the intervention [31]. Level 4, results, is the degree that targeted outcomes and changes in practice occur due to the learning intervention [25]. None of the studies evaluated this highest level of learning outcomes.

### Theoretical Frameworks of Microlearning

Although they were not found in every study, some theoretical frameworks were identified as foundational for microlearning. Banning’s theoretical framework used in Chuang and Tsao’s study of the effect of microlearning on nursing pharmacology students examined how students acquire, store, and retrieve knowledge and how it differentiated between reasoning styles [32]. Chuang and Tsao and Sichani et al also used the information processing theory (IPT) to guide their curriculum design [32,33]. Similar to Banning’s framework, the IPT examined how individuals acquire, store, and then retrieve knowledge (stimulus and response). According to the IPT framework, an external stimulus is held in the sensory register for a short time and transferred to the short-term memory and eventually to the long-term memory by a process of organizing, repeating, elaborating, and distributing practice [32]. As such, Chuang and Tsao’s design included organized pharmacology information delivered to nursing students 2 times per day via text [32]. This repetition significantly increased students’ memory of cardiovascular medications and supplied the study material for later referral. Using the IPT, Sichani et al sent questions via text that were relevant to the material covered in lectures during class; these students showed significant improvements in pre- and posttest scores compared with students who did not receive the texts [33].

Swartzwelder used the social learning theory to examine students’ perceptions of the use of texting and its effects on learning comprehension compared with email [34]. In her study, the students who received weekly questions via text reported increased interactivity, convenience, and critical thinking [34]. Wang et al also examined interactivity using Henri’s analytical model as a pedagogical guide [35,36]. Henri’s model contains 5 dimensions: participative, social, interactive, cognitive, and metacognitive, and this model has been used extensively by educators to assess the learning process of discussion forums [36]. Interactivity consists of communication of information, an initial response to the information, followed by an answer to the initial response [36]. The researchers utilized participation, social attendance, level of interaction, and cognitive skills to evaluate participant responses to cases posted on the social media site Weibo, a site similar to Twitter [35].

The just-in-time training (JITT) model is a teaching methodology that provides tailor-made, immediate, and focused training; it is well suited for application to microlearning [4,5]. This method, originally rooted in the automotive industry, has migrated to education [29] and can be used to provide immediate information when it is needed the most, specifically for health care students and providers at the point of care with a patient. This modality can also be applied in remote regions of the world where education resources and trained health care professionals are uncommon. In our review, this model was used to teach the application of wrist splints to medical students [29]. Providing the JITT video to learners immediately before they were required to perform the procedure decreased learning time and improved overall performance. This type of pedagogical concept may increase performance and safety at the patient bedside and in remote parts of the world [29]. A summary of the characteristics of the 17 articles reviewed is provided in Multimedia Appendix 3 [26-35,37-43].

### Characteristics of Microlearning

To understand the characteristics of microlearning in the 17 studies, we utilized Buchem and Hamelmann’s review of microlearning that posits the following 10 concepts: (1) learning context, (2) time spent, (3) content type, (4) content creation, (5) content aggregation, (6) content retrieval, (7) structure of the learning cycle, (8) target group, (9) learner’s role, and (10) learner participation [19]. Multimedia Appendix 4 illustrates the presence of these characteristics in each study reviewed. As seen, each of the studies addressed some of the 10 microlearning characteristics, whereas only 2 of the studies, those by Bledsoe et al and Diug et al, presented all 10 characteristics [27,30]. The last column shows that each article satisfies at least 40% of the 10 concepts identified in Buchem and Hamelmann’s review [19].

#### Learning Context and Time Spent

Buchem and Hamelmann differentiated microlearning from macrolearning, explaining that the former offered informal learning opportunities that take place outside of the traditional classroom [19]. Looking closely at the context of the microlearning interventions, we found that many of the microlearning activities were used as supplemental tools to didactic and established courses (14/17, 82%). For example, Kalludi et al gave 1 group of Indian dental students access to 12-min audiovisual podcasts after they attended regular lectures; the control group did not see the podcasts [38]. Students who saw the podcasts performed significantly better on a follow-up multiple-choice questionnaire than those in the control group (*P*=.021) [38].

Unlike formal learning, the time spent on microlearning ranges from a few seconds to 15 min, which offers flexible, personalized on-demand learning [19]. Cheng et al utilized a 3-min instructional video on short-arm volar splinting to provide JITT to medical students at a US children’s medical center [29]. Both Sichani et al and Swartzwelder sent out single multiple-choice questions daily to students via SMS texts [33,34], whereas Lameris et al utilized an app to provide students with practice questions that needed to be answered within 60 seconds [31]. All of our 17 studies featured the concept of shortened time spent—less than 15 min.

#### Content Type and Creation

Another important key to microlearning is narrowing the topic to a single definable idea, published in short form and accessible through the Web 2.0 via blog posts, wiki pages, permalinks, and hashtags [19]. For example, to improve patient safety and outcomes by enhancing nursing students’ knowledge of pharmacology, Chuang and Tsao utilized mobile phone SMS texts limited to 70 Chinese words (including the names, actions, clinical uses, side effects, and contraindications of cardiovascular drugs) [32]. A study by Evans addressed the challenging topic of embryology with 5- to 10-min audiovisual screencasts on the fertilization and development of embryos for medical students in the United Kingdom [28].

Microlearning is regarded as a deviation from traditional transfers of knowledge between a subject matter expert and learner; most microcontent is cocreated by end users utilizing Web 2.0 and other e-learning tools [19]. Although all of the studies used microcontent for their course development and delivery process, of our 17 studies, only 5 (29%) cocreated learning content with students. For example, Bledsoe et al used the social media platform Twitter as a collaborative educational environment where American graduate psychology students created unique hashtags based on common topics among their members and then used these hashtags to communicate and share information regarding the course’s research questions [27]. Diug et al also utilized Twitter as a pedagogical tool [30]. They required first-year biomedical students to identify a public health issue in their daily lives by posting a photo, image, or link to a journal article of interest via Twitter, which was then linked through a hashtag to the course. Similarly, different Chinese microblogging platforms have been used to facilitate learning for pharmacy students by sending informative push notifications and actively responding to these, while allowing students to work together with their group to address patient scenarios via group chats [35,42,43].

#### Content Aggregation and Retrieval

Multiple learning objectives, which divide up and rearrange content, are usually developed to establish the scope of formal learning. However, these pieces of information collectively represent an idea or topic and rely on each other for clarity and completeness [19]. Microlearning consists of self-contained ideas that can stand alone without necessary supplementation because of the narrowed and concentrated focus of a topic [19]. All of our 17 studies featured these self-contained concepts. For example, Lameris et al utilized an open-source HTML-based app to focus specifically on circulation and respiration concepts for Dutch biomedical students in a physiomics course [31]. Over a 4-week period, the students used the app to study short modules and complete practice questions, after which they took their final exam [31].

Most traditional topics, including those used in e-learning, can be retrieved via unique URLs that direct a user to a broad concept and a collection of objectives. However, the unique URL of microcontent allows for the smallest bits of information to be retrieved and linked together while still being able to stand on their own [19]. Buchem and Hamelmann contended that large bundles of information on the internet are often ignored, whereas small pieces of the whole are tagged and linked in ways that create new patterns, ideas, and meaning [19]. Of our 17 studies, only 6 (35%) reported the use of unique URLs for content retrieval. Examples include the use of the computer program TweetDeck [44], which allows students to organize tweets from the accounts they follow [27] and the development of links called *5 Minute Medicine* to address common patient disorders that internal medicine residents would face during their patient assessments [39]. These learning materials were uploaded to the website, which is now available through a YouTube channel [45].

#### Structure of the Learning Cycle and Target Group

The framework of traditional macrolearning, based on learning objectives, is usually organized in a hierarchical and sequential fashion, whereas the structure of microlearning is dynamic and fluid, based on the user’s self-directed learning through aggregation and modification [19]. One example is the use of microblogging, which allows learners to write and edit structured and strategic responses, thus generating perceptions of credibility and trust [46]. In our review, only 5 studies (29%, 5/17) espoused the concept of nonsequential learning using microblogging. As an example, Diug et al used Twitter to encourage students to reflect on their learning regarding public health issues by tweeting about their use of the game app *Dumb Ways to Die*, a public service campaign developed by the metro stations in Melbourne, Australia, to promote railway safety [30]. In Wang’s study, students were required to complete case studies in their groups and work together to address disease states, therapeutic goals, drug information, adverse drug events, drug interactions, monitoring plan, and patient education utilizing the Chinese microblogging platform Sina Weibo, which is similar to Twitter [35].

As Buchem and Hamelmann posited, the goals of microlearning are broader than the learning outcomes defined by content experts in traditional macrolearning; they focus more on the exploration of concepts and practical problem solving. Microlearning is appealing to self-directed learners who are drawn to the informal, flexible, and shortened learning activities that can be easily integrated into their lives [19]. All of the studies in our review targeted learners who are practical and in search of information and knowledge that can be applied immediately to boost their careers and confidence.

#### Learner’s Role and Learner Participation

The student’s role within microlearning is not one of consuming content in an effort to mirror an expert but to produce the learning content through social interaction and concept exploration [19]. By shifting to the role of a *prosumer* transforming from a consumer to a producer, learners are more motivated and feel a greater responsibility for the achievement of their own learning goals [19]. Sichani et al argued that self-directed learning was encouraged by the delivery of single answer or multiple-choice questions to Iranian medical students via SMS [33]. Wang et al found a significant difference in the scores of the students who actively responded to the WeChat push notifications from faculty compared with those who did not reply (*P*<.01) [42]. Interestingly, this prosumer concept was not a common guiding principle in any of our 17 studies, as evidenced by the small number of cases (n=6, 35%).

Finally, Buchem and Hamelmann postulated that macrolearning relies on the learner’s interaction with predetermined content, whereas microlearning focuses on the social interactions of the users to drive the creation and transfer of ideas [19]. This concept was well presented in a study by Bledsoe et al who created a collaborative learning environment by utilizing Twitter to address questions from graduate students in a research methodology online course [27]. In this study, 83% of the students agreed that being part of a group aided in their learning of research concepts and 86% agreed that they were learning important research components. Wang et al also reported that more than 60% of the students who used mobile messaging–based case studies (MMBC) agreed that MMBC helped develop their skills and knowledge, understand others’ viewpoints, and share their experiences [43]. Social interactions between learners, compared with learner-content interactions, were present in 5 (29%) of the 17 studies reviewed.

## Discussion

### Principal Findings

This scoping review identified the literature surrounding the use of microlearning as a pedagogical tool in health professions education, which led to the discussion of what is known about microlearning. The studies demonstrated that microlearning can improve performance and potentially increase safety in the clinical environment [29,30]. This potential is consistent with a wide range of previous research on microlearning and medical training [14,16] and continuing education in nursing [13,15]. Results from previous studies along with our findings highlighted how microlearning could be used as a refresher before performing skills that are infrequently used or when performing new skills without previous experience. This could also improve safety as skills that are very complicated could be rehearsed and not performed from memory. Students can review difficult content as many times as needed to reinforce their understanding or immediately before performing new or difficult procedures in clinical education. In addition, according to the cognitive load theory, the characteristics of microlearning, particularly microcontent and content retrieval, enable learners to have long-term memory of learning materials by constructing small structures repeatedly [47,48].

Microlearning methods take advantage of the fact that mobile device usage is virtually universal [5]. This environment is conducive to communication and collaboration according to many of the studies in this review [30,32,35,43]. By using social media, podcasts, texting, and SMS, teachers are moving the classroom to the students and changing how they communicate and study [5]. Podcasts have transported the classroom to the virtual world and provide new methods to disseminate learning materials to students. This flexibility and accessibility of information is harnessed by self-directed learning. In a similar vein, students reported that viewing a supplemental video after a live lecture helped them to better understand the material [38]. Podcasts can also help connect the content between lectures and textbooks [39,49]. Students have credited podcasts with increased satisfaction in knowledge acquisition, and they have cited convenience as a major advantage [32,38,39,41]. The potential uses for apps designed specifically to deliver microlearning material are growing [5].

Despite its known benefits, there are some downsides to microlearning. First, traditionalists may have a hard time learning emerging technologies while experiencing increased stress related to change [50]. As instructors must be comfortable with Web 2.0, they may be required to train on the devices or software used. Microlearning may require time-consuming development and labor-intensive lesson planning [27,40]. Freed et al also found that faculty are concerned with lecture recording, which may encourage students to take a passive role in the classroom [50]. If podcasting is for review and not associated with active learning activities in class, the student may just listen to the lecture when studying for an exam and fail to read and utilize other forms of independent study [51]. Another concern from faculty is about who owns materials such as podcasts or audiovisual lectures [52]. Faculty may not be aware that institutional policies consider teaching materials they develop as the property of the employer [52]. Podcasts are stored digitally and often recycled [39]. Thus, if recycled over an extended period, the information will be outdated, which could reflect poorly on instructors. Thus, it is necessary for universities and faculties to develop clearly defined guidelines about educational podcasts [52].

Microlearning relies on having network connectivity and interactivity. Himmelsbach suggested that technology in education may create a disconnect from social interactions [53]. As discussed in Wang et al’s study, some students believed that the collaborative learning was not effective, the quality of interaction was low, and it was hard to follow the stream of comments because of the large volume of interactions [35]. Students do not necessarily have equal access to technology [38]; therefore, faculty must ensure adequate access and support before implementing microlearning on technologies some students may not possess. In addition, there are subject areas that are too complicated for the use of microlearning alone. Poorly designed objectives can have less-than-desirable outcomes when using Web 2.0–based learning [54]. Faculty and student privacy may also be a concern with the adoption of social media in the classroom [55]. There is potential for information to be misconstrued regarding an individual, an institution, or the facts provided. To prevent learning bonds from being eroded, sound pedagogical principles and rules for respectful communication need to be in place.

### Limitations and Future Directions

This review has several limitations. First, the definition of microlearning is not universal, which limits the search terms and results. Studies that were included in the review utilized microlearning without mentioning the term or its definition. Thus, despite the extensive search, some literature may have been missed. Second, research was limited to English-language publications. According to the search results, more than half of the studies (9/17, 53%) were conducted in a country that uses languages other than English as the primary language. Therefore, there may be other microlearning studies that were not referenced because they were not in English. Finally, the younger age of the study participants might limit the ability to generalize the methods to older and less technologically savvy students.

Microlearning is a relatively new educational paradigm that has potential for both educators and students. Future research in this field should look at higher levels of learning outcomes from various microlearning modalities by designing studies that evaluate Kirkpatrick level 3 and 4 outcome measures. One characteristic of microlearning is that the learner is a prosumer and cocreator of content. This particular characteristic has not been applied widely in previous studies. Learners’ active engagement through their prosumer role and classroom interaction, combined with Web 2.0 and mobile technology, will allow health professions educators to provide more meaningful outcomes for students. Future studies should also incorporate larger and more diverse samples including traditional and adult learners with various degrees of technological ability. Future research might compare microlearning modalities and determine if one type of microlearning is more effective than another method.

### Conclusions

The aim of this review was to synthesize evidence on the use of microlearning in health professions education. As an education strategy, microlearning has the potential to change the way education is delivered to health professions students. Microlearning not only has the potential to change the way education is delivered to health professions students, but it is also a response to the novel methods that students learn, socialize, and communicate. By bringing the classroom to where students congregate and using methods based on theories of how the brain stores and retrieves information, microlearning can facilitate and enhance student learning. The findings of this scoping review will inform educational researchers, faculty, and academic administrators on the application of microlearning, pinpoint gaps in the literature, and help identify opportunities for instructional designers and subject matter experts to improve course content.

## Appendix

Multimedia Appendix 1 Search strategy.

Multimedia Appendix 2 Preferred Reporting Items for Systematic Reviews and Meta-Analyses extension for Scoping Reviews flowchart for the article search.

Multimedia Appendix 3 Summary of studies reviewed on microlearning in health professions education.

Multimedia Appendix 4 Presence of characteristics in each study (✓ concept was found in the study; — concept was not found in the study).

## Abbreviations

e-learning: electronic learning
IPT: information processing theory
JBI: Joanna Briggs Institute
JITT: just-in-time training
MMBC: mobile messaging–based case studies
SMS: short messaging service
