# Supplementary material for: Microlearning in Health Professions Education: Scoping Review
Source: JMIR Med Educ. 2019 Jul 23;5(2):e13997. doi: 10.2196/13997 (PMC6683654; doi:10.2196/13997)
Supplement: Multimedia Appendix 1 [file mededu_v5i2e13997_app1.docx]

## **Appendices**

**Appendix I – Search strategy**

**Database: PubMed**

| Set # | Search Query | Results |
| --- | --- | --- |
| 1 | (Microlearning[tw] OR "micro learning"[tw] OR microteaching[tw] OR "micro teaching"[tw] OR microlecture[tw] OR microlectures[tw] OR "micro lecture"[tw] OR "micro lectures"[tw] OR “micro content”[tw] OR microcontent[tw] OR microfeedback[tw] OR “micro feedback”[tw] OR microeducation[tw] OR “micro education”[tw] OR microskill[tw] OR "micro skill"[tw] OR microskills[tw] OR "micro skills"[tw] OR mircoformat[tw] OR microformats[tw] OR "micro format"[tw] OR "mirco formats"[tw] OR "just-in-time learning"[tw] OR "just-in-time training"[tw] OR "mlearning"[tw] OR "m-learning"[tw]) OR (("Learning"[Mesh] OR "Teaching"[Mesh] OR learn[tiab] OR learning[tiab] OR learns[tiab] OR "e-learning"[tiab] OR "elearning"[tiab] OR teaching[tiab] OR teach[tiab]) AND ("Cell Phone"[Mesh] OR “mobile applications”[mesh] OR "Text Messaging"[Mesh] OR "Blogging"[Mesh] OR “cell phone”[tiab] OR cellphone[tiab] OR “smart phone”[tiab] OR smartphone[tiab] OR iphone[tiab] OR android[tiab] OR ipad[tiab] OR tablet[tiab] OR mobile[tiab] OR ubiquitous[tiab] OR apps[tiab] OR app[tiab] OR podcast[tiab] OR podcasts[tiab] OR podcasting[tiab] OR texting[tiab] OR "text message"[tiab] OR "text messages"[tiab] OR "text messaging"[tiab] OR "short message service"[tiab] OR blog[tiab] OR blogs[tiab] OR blogging[tiab] OR "instant messaging"[tiab] OR "instant message"[tiab])) | 6,499 |
| 2 | "Education, Professional"[Mesh] OR "Schools, Health Occupations"[Mesh] OR "Students, Health Occupations"[mesh] OR "Faculty, Nursing"[Mesh] OR "Faculty, Medical"[mesh] OR "Faculty, Dental"[Mesh] OR ((Nursing[tiab] OR medical[tiab] OR dental[tiab] OR pharmacy[tiab] OR "public health"[tiab] OR "allied health"[tiab] OR chiropractic[tiab] OR midwifery[tiab] Or podiatry[tiab]) AND (education[tiab] OR school[tiab] OR schools[tiab] OR student[tiab] OR students[tiab] OR faculty[tiab])) | 466,464 |
| 3 | #1 AND #2 | 1,267 |
| 4 | #3 NOT (((teens[tiab] OR teen[tiab] OR teenager[tiab] OR adolescent[tiab] OR adolescents[tiab] OR adolescence[tiab] OR child[tiab] OR kid[tiab] OR kids[tiab] OR children[tiab] OR youth[tiab] OR “high school”[tiab] OR infant[tiab] OR newborn[tiab] OR adolescent[Mesh] OR child[Mesh] OR infant[Mesh])) NOT "Adult"[Mesh]) | 1,212 |
| 5 | #4 AND English[lang] | 1,134 |

**Database: CINAHL (via EBSCO)**

| Set # | Search Query | Results |
| --- | --- | --- |
| 1 | (TI(Microlearning OR "micro learning" OR microteaching OR "micro teaching" OR microlecture OR microlectures OR "micro lecture" OR "micro lectures" OR “micro content” OR microcontent OR microfeedback OR “micro feedback” OR microeducation OR “micro education” OR microskill OR "micro skill" OR microskills OR "micro skills" OR mircoformat OR microformats OR "micro format" OR "mirco formats" OR "just-in-time learning" OR "just-in-time training" OR "mlearning" OR "m-learning") OR AB(Microlearning OR "micro learning" OR microteaching OR "micro teaching" OR microlecture OR microlectures OR "micro lecture" OR "micro lectures" OR “micro content” OR microcontent OR microfeedback OR “micro feedback” OR microeducation OR “micro education” OR microskill OR "micro skill" OR microskills OR "micro skills" OR mircoformat OR microformats OR "micro format" OR "mirco formats" OR "just-in-time learning" OR "just-in-time training" OR "mlearning" OR "m-learning")) OR (((MH "Learning+") OR (MH "Teaching+") OR TI(learn OR learning OR learns OR "e-learning" OR elearning OR teaching OR teach) OR AB(learn OR learning OR learns OR "e-learning" OR elearning OR teaching OR teach)) AND ((MH "Cellular Phone+") OR (MH "Mobile Applications") OR (MH "Blogs") OR (MH "Instant Messaging") OR TI(“cell phone” OR cellphone OR “smart phone” OR smartphone OR iphone OR android OR ipad OR tablet OR mobile OR ubiquitous OR apps OR app OR podcast OR podcasts OR podcasting OR texting OR "text message" OR "text messages" OR "text messaging" OR "short message service" OR blog OR blogs OR blogging OR "instant messaging" OR "instant message") OR AB(“cell phone” OR cellphone OR “smart phone” OR smartphone OR iphone OR android OR ipad OR tablet OR mobile OR ubiquitous OR apps OR app OR podcast OR podcasts OR podcasting OR texting OR "text message" OR "text messages" OR "text messaging" OR "short message service" OR blog OR blogs OR blogging OR "instant messaging" OR "instant message"))) | 4,081 |
| 2 | MH "Education, Health Sciences" OR "Schools, Health Occupations" OR MH "Students, Health Occupations" OR MH "Faculty, Health Occupations" OR TI ((Nursing OR medical OR "allied health" OR chiropractic OR dental OR midwifery OR pharmacy OR "public health" OR podiatry) AND (education OR school OR schools OR student OR students OR faculty)) OR AB ((Nursing OR medical OR "allied health" OR chiropractic OR dental OR midwifery OR pharmacy OR "public health" OR podiatry) AND (education OR school OR schools OR student OR students OR faculty)) | 128,653 |
| 3 | #1 AND #2 | 523 |
| 4 | #3 NOT ((((TI (teens OR teen OR teenager OR adolescent OR adolescence OR adolescents OR child OR kid OR kids OR children OR youth OR “high school” OR infant OR newborn) OR AB (teens OR teen OR teenager OR adolescent OR adolescence OR adolescents OR child OR kid OR kids OR children OR youth OR “high school” OR infant OR newborn) OR MH "Adolescence" OR MH "Child")) NOT (MH "Adult+")) | 489 |
| 5 | #4 AND LA English | 455 |

**Database: ERIC (via EBSCO)**

| Set # | Search Query | Results |
| --- | --- | --- |
| 1 | DE "Microteaching" OR (TI(Microlearning OR "micro learning" OR microteaching OR "micro teaching" OR microlecture OR microlectures OR "micro lecture" OR "micro lectures" OR “micro content” OR microcontent OR microfeedback OR “micro feedback” OR microeducation OR “micro education” OR microskill OR "micro skill" OR microskills OR "micro skills" OR mircoformat OR microformats OR "micro format" OR "mirco formats" OR "just-in-time learning" OR "just-in-time training" OR "mlearning" OR "m-learning") OR AB(Microlearning OR "micro learning" OR microteaching OR "micro teaching" OR microlecture OR microlectures OR "micro lecture" OR "micro lectures" OR “micro content” OR microcontent OR microfeedback OR “micro feedback” OR microeducation OR “micro education” OR microskill OR "micro skill" OR microskills OR "micro skills" OR mircoformat OR microformats OR "micro format" OR "mirco formats" OR "just-in-time learning" OR "just-in-time training" OR "mlearning" OR "m-learning")) OR ((DE "Learning" OR DE "Electronic Learning" OR DE "Instruction" OR DE "College Instruction" OR TI(learn OR learning OR learns OR "e-learning" OR elearning OR teaching OR teach) OR AB(learn OR learning OR learns OR "e-learning" OR elearning OR teaching OR teach)) AND (DE "Handheld Devices" OR TI(“cell phone” OR cellphone OR “smart phone” OR smartphone OR iphone OR android OR ipad OR tablet OR mobile OR ubiquitous OR apps OR app OR podcast OR podcasts OR podcasting OR texting OR "text message" OR "text messages" OR "text messaging" OR "short message service" OR blog OR blogs OR blogging OR "instant messaging" OR "instant message") OR AB(“cell phone” OR cellphone OR “smart phone” OR smartphone OR iphone OR android OR ipad OR tablet OR mobile OR ubiquitous OR apps OR app OR podcast OR podcasts OR podcasting OR texting OR "text message" OR "text messages" OR "text messaging" OR "short message service" OR blog OR blogs OR blogging OR "instant messaging" OR "instant message"))) | 9,003 |
| 2 | DE "Medical Education" OR DE "Graduate Medical Education" OR DE "Nursing Education" OR DE "Pharmaceutical Education" OR DE "Allied Health Occupations Education" OR DE "Medical Schools" OR DE "Dental Schools" OR DE "Nursing Students" OR DE ”medical students” OR DE "Medical School Faculty" OR TI ((Nursing OR medical OR "allied health" OR chiropractic OR dental OR midwifery OR pharmacy OR "public health" OR podiatry) AND (education OR school OR schools OR student OR students OR faculty)) OR AB ((Nursing OR medical OR "allied health" OR chiropractic OR dental OR midwifery OR pharmacy OR "public health" OR podiatry) AND (education OR school OR schools OR student OR students OR faculty)) | 34,985 |
| 3 | #1 AND #2 | 201 |
| 4 | #3 NOT ((TI (teens OR teen OR teenager OR adolescent OR adolescence OR adolescents OR child OR kid OR kids OR children OR youth OR “high school” OR infant OR newborn) OR AB (teens OR teen OR teenager OR adolescent OR adolescence OR adolescents OR child OR kid OR kids OR children OR youth OR “high school” OR infant OR newborn) OR DE "Adolescents" OR DE "Children" OR DE "Early Adolescents")) NOT DE "Adults") | 178 |
| 6 | #5 AND LA English | 174 |

**Database: Embase**

| Set # | Search Query | Results |
| --- | --- | --- |
| 1 | (Microlearning:ti,ab OR 'micro learning':ti,ab OR microteaching:ti,ab OR 'micro teaching':ti,ab OR microlecture:ti,ab OR microlectures:ti,ab OR 'micro lecture':ti,ab OR 'micro lectures':ti,ab OR 'micro content':ti,ab OR microcontent:ti,ab OR microfeedback:ti,ab OR 'micro feedback':ti,ab OR microeducation:ti,ab OR 'micro education':ti,ab OR microskill:ti,ab OR 'micro skill':ti,ab OR microskills:ti,ab OR 'micro skills':ti,ab OR mircoformat:ti,ab OR microformats:ti,ab OR 'micro format':ti,ab OR 'mirco formats':ti,ab OR 'just-in-time learning':ti,ab OR 'just-in-time training':ti,ab OR 'mlearning':ti,ab OR 'm-learning':ti,ab) OR (('learning'/exp OR 'teaching'/de OR learn:ti,ab OR learning:ti,ab OR learns:ti,ab OR 'e-learning':ti,ab OR 'elearning':ti,ab OR teaching:ti,ab OR teach:ti,ab) AND ('mobile phone'/exp OR 'mobile application'/exp OR 'text messaging'/exp OR 'blogging'/exp OR 'podcast'/exp OR 'cell phone':ti,ab OR cellphone:ti,ab OR 'smart phone':ti,ab OR smartphone:ti,ab OR iphone:ti,ab OR android:ti,ab OR ipad:ti,ab OR tablet:ti,ab OR mobile:ti,ab OR ubiquitous:ti,ab OR apps:ti,ab OR app:ti,ab OR podcast:ti,ab OR podcasts:ti,ab OR podcasting:ti,ab OR texting:ti,ab OR 'text message':ti,ab OR 'text messages':ti,ab OR 'text messaging':ti,ab OR 'short message service':ti,ab OR blog:ti,ab OR blogs:ti,ab OR blogging:ti,ab OR 'instant messaging':ti,ab OR 'instant message':ti,ab)) | 9,223 |
| 2 | 'medical education'/exp OR 'paramedical education'/exp OR 'pharmacy school'/exp OR 'medical school'/exp OR 'health student'/exp OR 'continuing education'/exp OR 'faculty practice'/exp OR ((Nursing:ti,ab OR medical:ti,ab OR dental:ti,ab OR pharmacy:ti,ab OR 'public health':ti,ab OR 'allied health':ti,ab OR chiropractic:ti,ab OR midwifery:ti,ab OR podiatry:ti,ab) AND (education:ti,ab OR school:ti,ab OR schools:ti,ab OR student:ti,ab OR students:ti,ab OR faculty:ti,ab)) | 632,912 |
| 3 | #1 AND #2 | 2,083 |
| 4 | #3 NOT (((teens:ti,ab OR teen:ti,ab OR teenager:ti,ab OR adolescent:ti,ab OR adolescents:ti,ab OR adolescence:ti,ab OR child:ti,ab OR kid:ti,ab OR kids:ti,ab OR children:ti,ab OR youth:ti,ab OR 'high school':ti,ab OR infant:ti,ab OR newborn:ti,ab juvenile;ti,ab OR juvelines;ti,ab OR 'juvenile'/exp)) NOT 'adult'/exp) | 1,991 |
| 6 | #5 AND English:la | 1,162 |
| 7 | #6 AND [embase]/lim NOT [medline]/lim | 945 |

**Database: PsycINFO (via EBSCO)**

| Set # | Search Query | Results |
| --- | --- | --- |
| 1 | (TI(Microlearning OR "micro learning" OR microteaching OR "micro teaching" OR microlecture OR microlectures OR "micro lecture" OR "micro lectures" OR “micro content” OR microcontent OR microfeedback OR “micro feedback” OR microeducation OR “micro education” OR microskill OR "micro skill" OR microskills OR "micro skills" OR mircoformat OR microformats OR "micro format" OR "mirco formats" OR "just-in-time learning" OR "just-in-time training" OR "mlearning" OR "m-learning") OR AB(Microlearning OR "micro learning" OR microteaching OR "micro teaching" OR microlecture OR microlectures OR "micro lecture" OR "micro lectures" OR “micro content” OR microcontent OR microfeedback OR “micro feedback” OR microeducation OR “micro education” OR microskill OR "micro skill" OR microskills OR "micro skills" OR mircoformat OR microformats OR "micro format" OR "mirco formats" OR "just-in-time learning" OR "just-in-time training" OR "mlearning" OR "m-learning")) OR ((DE "Learning" OR DE "Electronic Learning" OR DE "School Learning" OR DE "Teaching" OR DE "Teaching Methods" OR TI(learn OR learning OR learns OR "e-learning" OR elearning OR teaching OR teach) OR AB(learn OR learning OR learns OR "e-learning" OR elearning OR teaching OR teach)) AND (DE "Mobile Devices" OR DE "Cellular Phones" OR DE "Text Messaging" OR DE "Blog" OR TI(“cell phone” OR cellphone OR “smart phone” OR smartphone OR iphone OR android OR ipad OR tablet OR mobile OR ubiquitous OR apps OR app OR podcast OR podcasts OR podcasting OR texting OR "text message" OR "text messages" OR "text messaging" OR "short message service" OR blog OR blogs OR blogging OR "instant messaging" OR "instant message") OR AB(“cell phone” OR cellphone OR “smart phone” OR smartphone OR iphone OR android OR ipad OR tablet OR mobile OR ubiquitous OR apps OR app OR podcast OR podcasts OR podcasting OR texting OR "text message" OR "text messages" OR "text messaging" OR "short message service" OR blog OR blogs OR blogging OR "instant messaging" OR "instant message"))) | 6,260 |
| 2 | DE "Medical Education" OR DE "Medical Internship" OR DE "Medical Residency" OR DE "Nursing Education" OR DE "Graduate Schools" OR DE "Graduate Students" OR DE "Medical Students" OR DE "Nursing Students" OR DE "Dental Students" OR TI ((Nursing OR medical OR "allied health" OR chiropractic OR dental OR midwifery OR pharmacy OR "public health" OR podiatry) AND (education OR school OR schools OR student OR students OR faculty)) OR AB ((Nursing OR medical OR "allied health" OR chiropractic OR dental OR midwifery OR pharmacy OR "public health" OR podiatry) AND (education OR school OR schools OR student OR students OR faculty)) | 83,756 |
| 3 | #1 AND #2 | 276 |
| 4 | #3 NOT ((TI (teens OR teen OR teenager OR adolescent OR adolescence OR adolescents OR child OR kid OR kids OR children OR youth OR “high school” OR infant OR newborn) OR AB (teens OR teen OR teenager OR adolescent OR adolescence OR adolescents OR child OR kid OR kids OR children OR youth OR “high school” OR infant OR newborn)) NOT (TI(adult or adults) OR AB(adult or adults)) | 249 |
| 5 | #5 AND LA English | 247 |

**Database: Education Full Text (H.W. Wilson)**

| Set # | Search Query | Results |
| --- | --- | --- |
| 1 | DE "Microlearning" OR DE "Microteaching"DE "Microlearning" OR (TI(Microlearning OR "micro learning" OR microteaching OR "micro teaching" OR microlecture OR microlectures OR "micro lecture" OR "micro lectures" OR “micro content” OR microcontent OR microfeedback OR “micro feedback” OR microeducation OR “micro education” OR microskill OR "micro skill" OR microskills OR "micro skills" OR mircoformat OR microformats OR "micro format" OR "mirco formats" OR "just-in-time learning" OR "just-in-time training" OR "mlearning" OR "m-learning") OR AB(Microlearning OR "micro learning" OR microteaching OR "micro teaching" OR microlecture OR microlectures OR "micro lecture" OR "micro lectures" OR “micro content” OR microcontent OR microfeedback OR “micro feedback” OR microeducation OR “micro education” OR microskill OR "micro skill" OR microskills OR "micro skills" OR mircoformat OR microformats OR "micro format" OR "mirco formats" OR "just-in-time learning" OR "just-in-time training" OR "mlearning" OR "m-learning")) OR ((DE "Learning" OR DE "Teaching" OR TI(learn OR learning OR learns OR "e-learning" OR elearning OR teaching OR teach) OR AB(learn OR learning OR learns OR "e-learning" OR elearning OR teaching OR teach)) AND (DE "Mobile apps in education" OR TI(“cell phone” OR cellphone OR “smart phone” OR smartphone OR iphone OR android OR ipad OR tablet OR mobile OR ubiquitous OR apps OR app OR podcast OR podcasts OR podcasting OR texting OR "text message" OR "text messages" OR "text messaging" OR "short message service" OR blog OR blogs OR blogging OR "instant messaging" OR "instant message") OR AB(“cell phone” OR cellphone OR “smart phone” OR smartphone OR iphone OR android OR ipad OR tablet OR mobile OR ubiquitous OR apps OR app OR podcast OR podcasts OR podcasting OR texting OR "text message" OR "text messages" OR "text messaging" OR "short message service" OR blog OR blogs OR blogging OR "instant messaging" OR "instant message"))) | 4,025 |
| 2 | DE "Graduate medical education" OR DE "Nursing education" OR DE "Associate degree nursing education" OR DE "Baccalaureate nursing education" OR DE "Continuing education of nurses" OR DE "Nursing education (Graduate)" OR DE "Nurse educators" OR DE "Nursing schools" OR DE "Nursing students" OR DE "Nursing teachers" OR DE "Pharmaceutical education" OR DE "Pharmacy colleges" OR DE "Paramedical education" OR DE "Education of physicians' assistants" OR DE "Physical therapy education" OR DE "Health occupations schools" OR DE "Chiropractic schools" OR DE "Dental schools" OR DE "Medical schools" OR DE "Public health schools" OR DE "Health occupations school faculty" OR DE "Health occupations students" OR DE "Continuing education of nurses" OR DE "Medicine -- Study & teaching (Continuing education)" OR TI ((Nursing OR medical OR "allied health" OR chiropractic OR dental OR midwifery OR pharmacy OR "public health" OR podiatry) AND (education OR school OR schools OR student OR students OR faculty)) OR AB ((Nursing OR medical OR "allied health" OR chiropractic OR dental OR midwifery OR pharmacy OR "public health" OR podiatry) AND (education OR school OR schools OR student OR students OR faculty)) | 17,649 |
| 3 | #1 AND #2 | 114 |
| 4 | #3 NOT ((TI (teens OR teen OR teenager OR adolescent OR adolescence OR adolescents OR child OR kid OR kids OR children OR youth OR “high school” OR infant OR newborn) OR AB (teens OR teen OR teenager OR adolescent OR adolescence OR adolescents OR child OR kid OR kids OR children OR youth OR “high school” OR infant OR newborn) OR DE "Teenagers" OR DE "Youth" OR DE "Adolescence" OR DE "Children" OR DE "High school students")) NOT DE "Adults") | 108 |
| 5 | #5 AND LA English | 108 |

**Database: ProQuest Dissertations & Theses Global**

| Set # | Search Query | Results |
| --- | --- | --- |
| 1 | (TI(Microlearning OR "micro learning" OR microteaching OR "micro teaching" OR microlecture OR microlectures OR "micro lecture" OR "micro lectures" OR “micro content” OR microcontent OR microfeedback OR “micro feedback” OR microeducation OR “micro education” OR microskill OR "micro skill" OR microskills OR "micro skills" OR mircoformat OR microformats OR "micro format" OR "mirco formats" OR "just-in-time learning" OR "just-in-time training" OR "mlearning" OR "m-learning") OR AB(Microlearning OR "micro learning" OR microteaching OR "micro teaching" OR microlecture OR microlectures OR "micro lecture" OR "micro lectures" OR “micro content” OR microcontent OR microfeedback OR “micro feedback” OR microeducation OR “micro education” OR microskill OR "micro skill" OR microskills OR "micro skills" OR mircoformat OR microformats OR "micro format" OR "mirco formats" OR "just-in-time learning" OR "just-in-time training" OR "mlearning" OR "m-learning")) OR (SU.EXACT (Learning) OR SU.EXACT(Teaching) OR TI(learn OR learning OR learns OR "e-learning" OR elearning OR teaching OR teach) OR AB(learn OR learning OR learns OR "e-learning" OR elearning OR teaching OR teach)) AND (SU.EXACT (smartphone) OR SU.EXACT (cellular telephone) OR SU.EXACT (text messaging) OR SU.EXACT(instant messaging) OR TI(“cell phone” OR cellphone OR “smart phone” OR smartphone OR iphone OR android OR ipad OR tablet OR mobile OR ubiquitous OR apps OR app OR podcast OR podcasts OR podcasting OR texting OR "text message" OR "text messages" OR "text messaging" OR "short message service" OR blog OR blogs OR blogging OR "instant messaging" OR "instant message") OR AB(“cell phone” OR cellphone OR “smart phone” OR smartphone OR iphone OR android OR ipad OR tablet OR mobile OR ubiquitous OR apps OR app OR podcast OR podcasts OR podcasting OR texting OR "text message" OR "text messages" OR "text messaging" OR "short message service" OR blog OR blogs OR blogging OR "instant messaging" OR "instant message")) | 5,146 |
| 2 | SU.EXACT(medical schools) OR SU.EXACT(medical residencies) OR SU.EXACT(dental schools) OR SU.EXACT(nursing schools) OR SU.EXACT(nursing education) OR (TI(Nursing OR medical OR "allied health" OR chiropractic OR dental OR midwifery OR pharmacy OR "public health" OR podiatry) AND TI(education OR school OR schools OR student OR students OR faculty)) OR (AB (Nursing OR medical OR "allied health" OR chiropractic OR dental OR midwifery OR pharmacy OR "public health" OR podiatry) AND AB(education OR school OR schools OR student OR students OR faculty)) | 28,520 |
| 3 | #1 AND #2 | 131 |
| 4 | #3 NOT ((TI (teens OR teen OR teenager OR adolescent OR adolescence OR adolescents OR child OR kid OR kids OR children OR youth OR "young adult" OR "young adults"OR high school OR infant OR newborn) OR AB (teens OR teen OR teenager OR adolescent OR adolescence OR adolescents OR child OR kid OR kids OR children OR youth OR "young adult" OR "young adults" OR high school OR infant OR newborn)) NOT (SU(adult) OR TI(adult OR adults) OR AB(adult OR adults))) | 33 |
| 5 | #5, Filters: English language | 33 |
