# Supplementary figures and images for: Microlearning in Health Professions Education: Scoping Review
Source: JMIR Med Educ. 2019 Jul 23;5(2):e13997. doi: 10.2196/13997 (PMC6683654; doi:10.2196/13997)

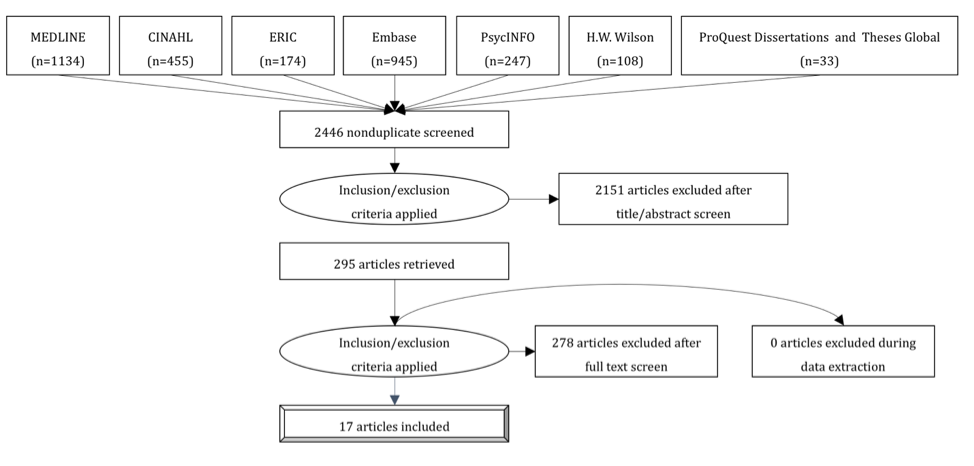

Supplement: Multimedia Appendix 2 [file mededu_v5i2e13997_app2.png]
