# Supplementary material for: Microlearning in Health Professions Education: Scoping Review
Source: JMIR Med Educ. 2019 Jul 23;5(2):e13997. doi: 10.2196/13997 (PMC6683654; doi:10.2196/13997)
Supplement: Multimedia Appendix 3 [file mededu_v5i2e13997_app3.docx]

**Appendix 3. Summary of studies reviewed on microlearning in health professions education.**

| Studies | Study purpose/theoretical underpinning if described | Study design/sample/course content | Key findings | Kirkpatrick levels |
| --- | --- | --- | --- | --- |
| Ball et al [26] | To study the effects of a video podcast-based violence education program on improvement of medical students’ knowledge and confidence in identifying and responding to violence | Quasi-experimental (quantitative); n=141 (fourth-year medical students during emergency medicine clerkship); and violent person management (VPM) | Students who viewed the video podcast showed significant improvement in knowledge (*P*<.01), and the mean score of knowledge increased from 4.07(SD 1.24) to 5.82 (SD 1.4; *P*<.05); the knowledge mean score of students who did not watch the video fell from 4.33 (SD 1.106) to 4.13 (SD 1.48). The students’ confidence in identifying potential violence was not significantly changed (*P*=.207), whereas their confidence in responding to violence has significantly improved (*P*=.004). About 60% of students agreed that the information in the video is helpful; 80% of them agreed that the lecture was professionally relevant to students; and 70% of them agreed that the delivery method of the video was appropriate. | Level 1: students’ responses to the podcast (helpfulness, relevance, appropriateness).  Level 2: student’s knowledge of and confidence in VPM |
| Bledsoe et al [27] | To explore the use of Twitter and hashtag in collaborative online educational environments. Personal learning networks—social constructivism; process-oriented learning; and grounded theory approach | Descriptive (mixed methods); n=62 (graduate students in a research methodology online course); and research methodology | Students commented that the access to information via Twitter was wider and easier. Students stated that the advantage of Twitter as a communication tool was *in real time*, and the disadvantage was *confining*. Some students reported that Twitter helped increase the class engagement, whereas others were concerned that the tweeting process was confusing and time-consuming. In addition, 50% of the participants reported enjoying the Twitter experience and 83% agreed that being part of a group aided in their learning of research methods. | Level 1: students’ feedback about the Twitter experience (access to information, communication, classroom engagement, overall experience) |
| Cheng et al [29] | To evaluate the utility of Just-in-time training (JITT) for teaching medical students the short-arm (SA) volar splinting technique; JITT model | Randomized controlled trial (quantitative); n=41 (control: 19; experimental: 22); and SA volar splinting with plaster | Watching a 3-min instructional video immediately before performing SA volar splint application with plaster (JITT experiment group) was associated with shorter self-instruction, shorter (preparation) times, higher splint assessment raw scores, and a higher rate of successful splint application in comparison with reading through medical textbooks (control group). A student’s overall collection of knowledge, based on years of training, was not significant compared with the presence of the JITT intervention in terms of learning efficiency or splinting success. | Level 2: splinting application, splinting time, assessment, and preparation, and completion |
| Chuang and Tsao [32] | To evaluate the effect of mobile phone short message services (SMS) on nursing students’ medication knowledge: Banning’s theoretical framework (acquisition of knowledge, storage and utilization of knowledge, and style of reasoning); information processing theory (how to receive new information, store it, and then recall it) | Quasi-experimental (quantitative); n=111 (control: 56; experimental: 55); cardiovascular medication | The average score on the medication knowledge questionnaire (MKQ) in the experimental group increased from 8.92 points at baseline to 13.46, 13.33, and 12.4 points at 1 week, 2 weeks, and 4 weeks, respectively, after the intervention. The knowledge (MKQ) score was significantly different between the control and intervention groups over time. The mean satisfaction level with the SMS intervention in the intervention group was 3.68 on a scale of 1-5 (1=very dissatisfied; 5=very satisfied). Nursing students who had greater satisfaction with the intervention scored higher in medication knowledge (r=.29, *P*= .04). | Level 1: satisfaction with this learning method.  Level 2: medication knowledge |
| Diug et al [30] | To study the use of Twitter as a pedagogical tool for student engagement with staff, peers, and course content | Descriptive study (quantitative); n=297 (first-year undergraduate students in a public health course); public health | Students’ self-reported attitudes were positive toward the use of social media as a learning tool; students agreed that the tasks provided greater access to staff and increased awareness of public health in their daily lives. Those who completed the task expressed a lack of peer collaboration, whereas those who did not complete the task expressed an increased collaboration among peers. Students who completed the task had significantly higher end-of-semester grades when compared with those who did not complete the task. There was no significant difference in end-of-semester grades when comparing students who had previously used social media with those who had no previous use of social media. The most common accessing method to social media was a mobile device (49.1%). | Level 1: awareness of public health; access to staff (accessibility); and engagement.  Level 2: Twitter task; peer-collaboration.  Level 3: end-of-semester grade |
| Evans (2011) [28] | To examine the use of and student reaction to a set of screencasts introduced to accompany embryology lectures | Descriptive study (quantitative); n=112 (second-year medical students learning embryology); embryology | Some students downloaded a single screencast up to 16 times. The most popular time and day for downloading was 8 pm and Wednesday, and the highest number of downloads was made on the day before the written examination. Most students viewed the screencasts favorably in terms of usefulness to their learning, good support to lectures, and effective review aid. Students scored well in the growth and development theme of the exam and demonstrated high average levels of knowledge and understanding. | Level 1: student satisfaction with quality and usefulness of screencasts; student usage.  Level 2: student attainment in the end-of-module written examination |
| Kalludi et al [37] | To assess the efficacy of audio podcasts as a supplementary of teaching and learning aid and to describe the students’ attitudes and perceptions regarding the utility of podcasts | Quasi-experimental (quantitative); n=80 (control: n=40; experimental: n=40); dental course | Students in the experimental group performed significantly better in a multiple-choice question (MCQ) test than students who were not supplemented with audio podcasts (*P*=.000). Overall, 91% of the students found the podcasts useful, as they could listen to lecture content repeatedly at their own convenience; 74% of the students had a positive attitude toward using the podcast as a supplementary tool for their learning; 76% of the students believed that including podcasts in the course curriculum would help them perform better in their exam; and 63% of the students felt that the absence of images and diagrams in podcasts was a disadvantage. | Level 1: students’ attitude toward audio podcasts.  Level 2: MCQ test |
| Kalludi et al [38] | To assess the efficacy of video podcasts as a supplementary teaching aid and to describe the students’ attitude and perceptions regarding the utility of podcasts | Quasi-experimental (quantitative); n=100 (control: n=54; experimental: n=46); dental course | A significant gain in the intervention group was noted when compared with the control group (*P*=.021); 89% of the students agreed that video podcasts might be useful, as it would enable them to view slides and hear the lectures repeatedly. Some students (71%) felt that video podcasts might have a disadvantage as a computer or a laptop may be required to view video podcasts, which may not always be feasible; 70% of students agreed that video podcasts are not a convenient learning tool as they might face technical difficulties. | Level 1: students’ attitude toward video podcasts.  Level 2: MCQ test |
| Lameris et al [31] | To determine the effect of formative testing using an internet-based app on study behavior and performance of medical students. Game-based learning: the use of games to enhance the learning experience | Correlational (quantitative); n=461 (336 medicine students; 125 biomedical science students); circulation and respiration | The app was well received by students; students scored the app with a grade of 7.3 (SD 1.0) out of 10; 59% of the students would like the app to be implemented in future courses; 34% of the respondents stated that the app positively affected their study behavior, and 54% stated the app helped them in their exam preparations. App users obtained significantly higher grades on the final exam of the course compared with nonusers (*P*<.001). The number of hours students spent on studying increased gradually during the 4-week course for nonusers as well as for moderate (*P*=.036) and intensive users (*P*<.01); 22% of the students failed to pass their final exam, and the percentage of students failing for the exam correlates with the use of the app. | Level 1: evaluation of the app.  Level 2: study performance in the final exam.  Level 3: study behavior |
| Narula et al [39] | To investigate the efficacy, usability, and time-effectiveness of 5 Minute Medicine (5MM) video clip podcasts as an educational tool for clinical clerks before assessing patients on call. Student-centered learning—access anytime, anywhere; to pause, rewind, and record; and to view the resource multiple times | Quasi-experimental (quantitative); n=73 (control: 37; experimental: 36); internal medicine (eg, atrial fibrillation and acute renal failure) | The majority of respondents ranked the 5MM superior to teaching from junior and senior medicine residents on their medicine rotation; 87% of the respondents selected 5MM as their preferred resource; 74% of the students were comfortable or very comfortable after the use of videos with their approach to a symptom or disorder (vs 60% in the control group). The amount of time required to prepare before patient assessment was significantly less for students in the experimental group than for those in the control group (11.1 min vs 19.2 min). The majority of students in the experimental group agreed that it was an effective way (97%), appropriate for their level of training (77%), and time-effective way (91%) to learn about symptoms/diseases. | Level 1: student reactions to video podcasts as a learning tool: usefulness, effectiveness, level of comfort, time-efficacy, appropriateness, etc) |
| Prakash et al [40] | To analyze the perceptions of medical students toward short-duration podcasts and self-learning | Descriptive study (mixed-methods); n=94 (first-year medical students in a biochemistry course (learning fat-soluble vitamins, heme metabolism, and disorders of hemoglobin); biochemistry (eg, fat-soluble vitamins and heme metabolism) | Overall, podcasts were well received, and 86% (n=76) of students perceived 3-MinuTe Lessons (MTLs) as useful. Time of podcasts: 37 students (49%) perceived the length was optimal, and an equal proportion suggested the duration can be increased, preferably to 5 min. Quality of podcasts: ease of downloading (lowest score), use of simple, clear language (highest score). The usefulness of podcasts: motivation obtained to read the topic (lowest score), helping to prepare for the test (highest score). Ease of downloading was rated lower among students who accessed the 3MTLs on phones compared with computers; the major feedback concerning the format of podcasts was toward the improvement of audio and video quality. Overall, average test scores of the 3MTL group were not significantly different from either assessment that immediately preceded or succeeded groups. Pairwise comparisons (Test3MTL vs TestPreceded; Test3MTL vs TestSucceeded) were statistically significant: Heavy users showed that benefit was consistently observed only amongst *above-average* performers. A few students described 3MTLs helpful in gaining an overview of the topic and studying. 3MTLs were helpful in improving their understanding of the topic, clarifying concepts, and focusing on important points. Teacher’s voice-over in the background was reassuring and useful in learning and made them lively. | Level 1: student responses on the usefulness and helpfulness of the podcasts (satisfaction related to learning, engagement, and quality).  Level 2: knowledge on anatomy via written assessment (a written test for a maximum score of 30 and absolute score obtained by students); essay and short answer descriptive type, testing a combination of factual recall and application-based knowledge |
| Richardson et al [41] | To investigate the usefulness of text messages in an undergraduate nursing anatomy course; spacing learning theory | Descriptive study (quantitative); n=246 (first-year nursing students in an anatomy and physiology course); anatomy and physiology | Of the 246 students, 17% signed up to receive text messages regarding concepts of anatomy, and the remaining 83% chose to receive the same message through the email default pathway; 59% of text users indicated that short messages were very useful, and 41% rated the messages as moderately useful; the percentages among email users were 41% and 50%, respectively. Both text users and email users preferred either material previously covered or a mixture of previously covered materials and future materials. The majority in each group of users had no preference which time of day they receive text messages. The second most preferred time was the evening (6 pm-12 am) among text users and afternoon (12 pm-6 pm) among email users. The majority of text users reported 5 or more texts per week was appropriate, while email users preferred 3 messages per week. All text users (100%) agreed that texts should be incorporated into the anatomy curriculum, whereas 82% of the email users viewed it as a feature that should be continued for future classes. The feedback from text users was positive. However, there was still a significant portion (83%) of the student population that chose to receive the default emails rather than text messages. | Level 1: students’ opinion toward the usefulness of text messages in the course.  Level 3: behavioral change (self-reporting: the text message reminder was important to students because it initiated their study of anatomy) |
| Sichani et al [33] | To determine the effect of sending educational questions through SMS on academic achievement and satisfaction of medical students and compare that with lecture teaching; information processing theory | Quasi-experimental (quantitative); n=47 (ninth-semester medical students in a urology course): kidney tumor (SMS) versus bladder tumor (lecture session); urology | There was no difference between pretest scores (*P*=.38, *t*=−.880), but posttest and delay test scores were significantly higher in the SMS learning group than in the lecture group (*P*<.05, *t*=2.480) and (*P*<.05, *t*=2.24); 37 (79%) students were not satisfied with the SMS learning method. Most dissatisfaction with the consequences of SMS learning had no impact on enhancing useful study hours. | Level 1: students’ satisfaction with an SMS learning experience.  Level 2: knowledge of kidney and bladder tumors |
| Swartzwelder [34] | To determine the effect of texting in an online class on the learning experience; social learning theory | Descriptive study (mixed methods); n=117 (undergraduate nursing students in an online mental health course: control n=61; experimental n=56); online mental health | There was a significant difference in the learners’ perception of learning when texting was used as an instructional tool (*P*=.031). There was no significant difference in postintervention mean scores (grade) between the control and experimental groups (*P*>.05). Three major emerging themes of perception of learning among students and instructors were interactivity, convenience, and critical thinking. | Level 1: students’ satisfaction with WeChat.  Level 2: perceived learning (attitude)—students’ perceptions of learning (relevance, reflection, interactivity, tutor support, peer support, and interpretation).  Level 2: students’ knowledge on mental health via written assignments. Level 3: final grade |
| Wang et al [35] | To determine students’ attitudes toward microblog-based case studies (MBC) in a pharmacotherapy class. Henri’s analytical model: used to study the learning process of computer-mediated discussion groups informal educational settings (consisting of 5 dimensions: participative, social, interactive, cognitive, and metacognitive dimension) | Descriptive study (quantitative); n=126 (fourth-year pharmacy students in a pharmacotherapy introduction class); introduction to pharmacotherapy | Despite the recommendation to make a separate account, students preferred to use their own account (only 26% set up a new account). Cognitive skills: percentages for messages of elementary clarification, in-depth clarification, and inference were 27.9%, 31.9%, and 25%, respectively. The social presence, as well as the quality of interactions and messages, was low. No inappropriate posts were identified; 91% of respondents indicated that MBC helped them to share information with others. The majority of students agreed that MBC improved communication (more than 80%) and increased the amount of interaction (70%); more than 50% students found value in reading other students’ messages; 25% students believed the collaborative learning was not effective, 22% indicated the quality of interaction was low, and some reported it was hard to follow the stream of comments because of the large volume of interactions. | Level 1: satisfaction; preferences, participation, interactivity. Level 2: attitude−students’ perceived learning, sense of community (social presence). Level 2: cognitive skills (elementary clarification, in-depth clarification, inference, judgment, and application of strategies) via care plan |
| Wang et al [42] | To describe a 2-semester-long trial of WeChat on its student-centered teaching-learning communication; self- regulated learning | Descriptive study (mixed-methods); sample size not described (medical students in biochemistry and cellular biology course); biochemistry and cellular biology | Four most common aspects of the WeChat that need improvement were: after-class study materials, simulation exercises, answers to questions and feedback, and research progress relevant to the course. There was a significant increase in scores in the biochemistry and cellular biology course final exam with the WeChat app than without the app (*P*<.01). Students who gave effective feedback on WeChat achieved a significant increase in scores on final exams than those who ignored WeChat. No students ignored the push message: 75% of students responded and gave effective feedback. The students who worked in teams performed better and displayed enhanced creative thinking skills, and it was very convenient for international students to understand and not miss a message; 97.22% of users were satisfied with using WeChat. | Level 1: students’ satisfaction and engagement level with WeChat (ie, WeChat utility, effective feedback, and teacher-student interaction).  Level 2: after-class study; simulation exercises (successful in preparing for and reviewing lessons). Level 3: final grade |
| Wang et al [43] | To examine the attitudes of students toward the case studies based on mobile messaging‐based case (MMBC) studies in a pharmacotherapy class; case-based teaching; computer-supported collaborative learning | Descriptive study (mixed-methods); n=120 (fourth-year pharmacy students in a pharmacotherapy introduction class); introduction to pharmacotherapy | More than 70% of the students agreed MMBC helped develop skills and knowledge. More than 60% of the students felt that MMBC helped them understand others’ viewpoints and share their experience and knowledge. More than half of students perceived MMBC “promotes online interaction,” “facilitates communication,” and “motivation and stimulation.” The majority of students preferred MMBC to traditional case studies. | Level 1: satisfaction, preferences, participation, and interactivity.  Level 2: students’ attitudes toward MMBC and knowledge (disease state information, therapeutic goals, drug information, etc) |
