# Supplementary material for: Microlearning in Health Professions Education: Scoping Review
Source: JMIR Med Educ. 2019 Jul 23;5(2):e13997. doi: 10.2196/13997 (PMC6683654; doi:10.2196/13997)
Supplement: Multimedia Appendix 4 [file mededu_v5i2e13997_app4.docx]

**Appendix 4. Presence of characteristics in each study (**🗸 **concept was found in the study; — concept was NOT found in the study).**

| Studies | Concept | | | | | | | | | | Statistics, n (%) |
| --- | --- | --- | --- | --- | --- | --- | --- | --- | --- | --- | --- |
|  | Learning context: supplemental use | Time spent: <15 min | Content type: micro content | Content creation: cocreation | Content aggregation: self-contained unit | Content retrieval: unique URL | Structure of learning cycle: nonsequential | Target group: learners aiming at solving practical problems | Learner’s role: prosumer | Learner’s participation: social interaction |  |
|  |  |  |  |  |  |  |  |  |  |  |  |
| Ball et al [26] | — | 🗸 | 🗸 | — | 🗸 | 🗸 | — | 🗸 | — | — | 5 (50) |
| Bledsoe et al [27] | 🗸 | 🗸 | 🗸 | 🗸 | 🗸 | 🗸 | 🗸 | 🗸 | 🗸 | 🗸 | 10 (100) |
| Cheng et al [29] | — | 🗸 | 🗸 | — | 🗸 | — | — | 🗸 | — | — | 4 (40) |
| Chuang and Tsao [32] | 🗸 | 🗸 | 🗸 | — | 🗸 | — | — | 🗸 | — | — | 5 (50) |
| Diug et al [30] | 🗸 | 🗸 | 🗸 | 🗸 | 🗸 | 🗸 | 🗸 | 🗸 | 🗸 | 🗸 | 10 (100) |
| Evans [28] | 🗸 | 🗸 | 🗸 | — | 🗸 | 🗸 | — | 🗸 | — | — | 6 (60) |
| Kalludi et al [37] | 🗸 | 🗸 | 🗸 | — | 🗸 | — | — | 🗸 | — | — | 5 (50) |
| Kalludi et al [38] | 🗸 | 🗸 | 🗸 | — | 🗸 | — | — | 🗸 | — | — | 5 (50) |
| Lameris et al [31] | 🗸 | 🗸 | 🗸 | — | 🗸 | — | — | 🗸 | — | — | 5 (50) |
| Narula et al [39] | 🗸 | 🗸 | 🗸 | — | 🗸 | 🗸 | — | 🗸 | — | — | 6 (60) |
| Prakash et al [40] | 🗸 | 🗸 | 🗸 | — | 🗸 | 🗸 | — | 🗸 | — | — | 6 (60) |
| Richardson et al [41] | 🗸 | 🗸 | 🗸 | — | 🗸 | — | — | 🗸 | — | — | 5 (50) |
| Sichani et al [33] | — | 🗸 | 🗸 | — | 🗸 | — | — | 🗸 | 🗸 | — | 5 (50) |
| Swartzwelder [34] | 🗸 | 🗸 | 🗸 | — | 🗸 | — | — | 🗸 | — | — | 5 (50) |
| Wang et al [35] | 🗸 | 🗸 | 🗸 | 🗸 | 🗸 | — | 🗸 | 🗸 | 🗸 | 🗸 | 9 (90) |
| Wang et al [42] | 🗸 | 🗸 | 🗸 | 🗸 | 🗸 | — | 🗸 | 🗸 | 🗸 | 🗸 | 9 (90) |
| Wang et al [43] | 🗸 | 🗸 | 🗸 | 🗸 | 🗸 | — | 🗸 | 🗸 | 🗸 | 🗸 | 9 (90) |
| Statistics, n (%) | 14 (82) | 17 (100) | 17 (100) | 5 (29) | 17 (100) | 6 (35) | 5 (29) | 17 (100) | 6 (35) | 5 (29) | 17 (100) |
